# Spelling out the roles of individual nucleoporins in nuclear export of mRNA

**DOI:** 10.1080/19491034.2022.2076965

**Published:** 2022-05-20

**Authors:** Mark Tingey, Yichen Li, Wenlan Yu, Albert Young, Weidong Yang

**Affiliations:** a Department of Biology, Temple University, Philadelphia, Pennsylvania, USA; b Department of Genetics, Yale School of Medicine, Yale University, New Haven, Connecticut, USA

**Keywords:** Nucleocytoplasmic transport, super-resolution light microscopy, mRNA biology

## Abstract

The Nuclear Pore Complex (NPC) represents a critical passage through the nuclear envelope for nuclear import and export that impacts nearly every cellular process at some level. Recent technological advances in the form of Auxin Inducible Degron (AID) strategies and Single-Point Edge-Excitation sub-Diffraction (SPEED) microscopy have enabled us to provide new insight into the distinct functions and roles of nuclear basket nucleoporins (Nups) upon nuclear docking and export for mRNAs. In this paper, we provide a review of our recent findings as well as an assessment of new techniques, updated models, and future perspectives in the studies of mRNA’s nuclear export.

## Introduction

### The structure and function of the NPC

Nuclear pore complexes (NPCs) are massive multiprotein assemblies embedded within the nuclear envelope (NE). With a molecular mass of 66 MDa in *Saccharomyces cerevisiae* [1] to 125 MDa in *Xenopus oocyte* [2], the NPCs are among the largest and most complex protein structures in eukaryotic cells. They mediate the bidirectional transportation of proteins and ribonucleoparticles between the nucleus and the cytoplasm, which is indispensable for cellular functions. Therefore, associations and correlations between human diseases and NPC functions have been well documented in the past two decades, including immune diseases, viral infections, neurological diseases, cardiovascular disorders, and cancer [3–5].

The NPCs are composed of approximately 30 distinct proteins highly conserved in function from budding yeast to humans called nucleoporins (Nups). Nups can be classified into various groups according to their relative localization within the NPC, structural folds, or sequence motifs [6,7]. One of the most used classification systems for Nups separates them into three groups: (1) Transmembrane Nups with a transmembrane domain that anchoring the NPC to the NE; (2) Scaffold/structural Nups that form the predominant physical structure of the pore; and (3) Phenylalanine-glycine (FG) Nups that are essential for maintaining the permeable barrier of the NPC.

Transmembrane Nups are classified as such due to the transmembrane helices that anchor the Nup to the NE. This group of Nups also interacts with other non-membrane Nups, allowing the assembly of stable NPCs. Previous research indicates that double deletion of membrane Nups cause NPC mislocation and abnormal morphology in yeast [8], while transmembrane nucleoporin Ndc1 in vertebrates is required for NPC assembly [9,10], highlighting the structural and functional significance of membrane Nups.

Structural Nups, also referred to as scaffold Nups, are essential for the scaffold formation in NPCs and can be recognized by their unique α-solenoid or β-propeller folds[11]. They interact both with FG-Nups and membrane Nups, providing mainly structural support for NPCs. Approximately half of the characterized Nups can be categorized into this group. These scaffold Nups are concentrated at the nuclear ring, cytoplasmic ring, central spoke ring of the NPC, and linkers between each ring. Among all scaffold Nups, the Y- (or Nup84) complex has been well characterized. It contains seven to ten highly conserved proteins, forming a highly branched Y shape structure [12,13]. Analysis has shown that the Y-complex and the outer coat of the COPII vesicle share structural elements and interactions, suggesting a common ancestry between NPCs and vesicles [14,15].

The remaining one-third Nups contain tandem repeats of phenylalanine-glycine (FG repeats). Thus, they are classified as FG-Nups. The FG repeats regions are natively unfolded, forming selective barriers in the central channel of the NPC [16]. The function of FG-Nups within the cell are multivaried and critical including roles in import and export, mitosis, DNA repair and gene expression regulation [17,18]. Of particular interest is the role of FG-Nups in nuclear import and export, which is twofold. First, FG-Nups are directly responsible for the formation of the selectively permeable barrier that inhibits the passive diffusion of macromolecules above the diffusion limits of 40–60 kDa [19,20]. Second, FG-Nups bind to nuclear transport factors, promoting facilitated diffusion for selected molecules [21–23]. Studies have shown that lining [1]transport-factor-binding FG-Nups in artificial nanopores is sufficient for selective transport, highlighting the importance of FG-Nups in selective barrier formation in the NPC[24].

### Sub-regions of NPC and their notable roles in nucleocytoplasmic transport

As evaluated by electron microscopy (EM) reconstructions, the structure of vertebrate NPCs indicates that they are composed of an octagonal central ring-spoke assembly that is ∼40 nm in length with an internal diameter of ∼50 nm and external diameter of ∼120 nm. On the cytoplasmic face of the NPC, a cytoplasmic ring moiety with eight ∼50 nm cytoplasmic filaments is attached to the central framework. On the nuclear face of the NPC, the central framework is connected to the ∼75 nm nuclear basket, an NPC sub-region containing the nuclear ring moiety and eight nucleoplasmic filaments organized into a basket-like structure projecting toward the nuclear interior [25–30]. The central ring-spoke assembly is sandwiched by the cytoplasmic ring moiety and the nuclear basket, forming a functional pore for nucleocytoplasmic transportation. These dimensions are roughly true for all NPCs; however, there are minor differences present within the NPC that can distinguish the NPC of one species from another. Notably, a Cryo-Electron Tomography study by Maimon and colleagues demonstrated that the human NPC is structurally distinct from lower order eukaryote NPCs [28].

### The FG-Nups on the cytoplasmic side

**Figure 1.**
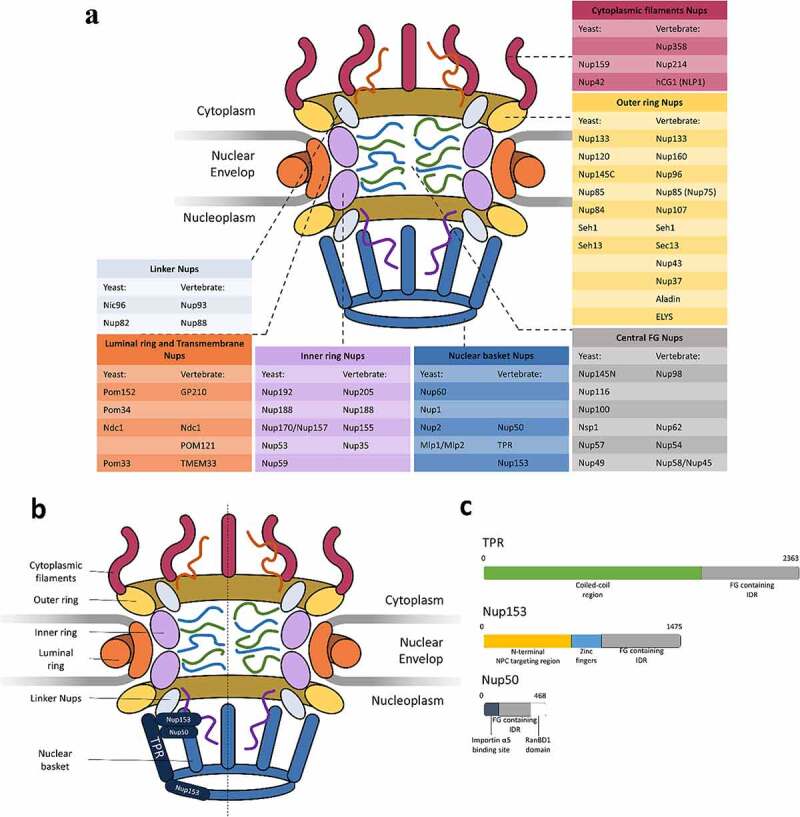
**Nuclear basket Nups**. (a) schematic illustration of overall structure of the NPC in yeast and vertebrate [78,208,209]. (b) schematic overview of nuclear basket Nup organization of the NPC in vertebrates. (c) relative sizes of nuclear basket Nups annotated with regions of note.

The FG-Nups on the cytoplasmic side of the NPC mainly contribute to nucleocytoplasmic transport. There are three FG-Nups mainly found on the cytoplasmic side of the vertebrate NPC, which are Nup214, hCG1(NLP1/CG1), and Nup358 (Figure 1a). Previous studies have linked messenger ribonucleoprotein (mRNP) remodeling with cytoplasmic FG regions. Double deletion of those regions in Nup42 and Nup159, which are yeast homologs of Nup214 and hCG1, cause a reduced capacity for mRNP remodeling during export [31]. This reduction was rescued by fusing the Nup42 FG domain to the C-terminus of RNA export mediator Gle1, suggesting FG domains target the mRNP to Gle1 and Dbp5 for mRNP remodeling at the NPC. Studies also showed that FG-repeat sequences within the extreme C-terminal end of Nup214 are essential for the interaction with nuclear export proteins Crm1 [32] and TAP, also called NXF1 [33], highlighting the significance of FG-regions on the cytoplasmic side of the NPC. Interestingly, the FG regions of hCG1 do not seem to interact with many export factors. It has been experimentally demonstrated that hCG1 interacts with CRM1 [34] and Gle1 [35] via non-FG regions.

Nup358 is the largest known Nup with a molecular mass of 358 kDa found only in vertebrate NPCs and unlike many Nups, does not have a yeast homolog. It is the major component of the cytoplasmic filaments, containing various structural regions including FG repeats, Ran GTP binding sites, zinc fingers, a cyclophilin A homologous domain, and a leucine-rich region [36]. Variations in import were observed in some research when the N-terminal leucine-rich region was fused with 200 amino acid sequences containing FG repeats, suggesting the involvement of FG repeats on efficient mRNA export [37]. However, due to the structural complexity and highly segmented FG regions, no conclusive explaination has been provided linking FG regions of Nup358 with nucleocytoplasmic transportation.

### The central ring-spoke assembly

The central ring-spoke assembly is the most complicated sub-region of the NPC, containing more than 20 Nups (Figure 1a). As the name indicates, the assembly contains eight identical protomer units referred to as ‘spokes,’ which were first identified and described via EM observation [38]. The eight spokes project radially from the NE membrane and surrounding a central tube of channel Nups. They also connect radially to form several concentric rings: the outer pore ring, inner pore ring, and the luminal ring.

The luminal ring (LR), also referred to as the membrane ring in some literature [39], resides in the NE lumen and surrounds the NPC at the site of membrane fusion [25]. Due to its unique localization, the LR is speculated to be composed of transmembrane Nups. Although there are four known vertebrate Nups with transmembrane helixes, GP210 is the only significant component of LR that contributes approximately 90% of its mass [25,40]. The molecular architecture of the LR for vertebrate NPCs has been reported recently using cryo-electron tomography and cryo-EM analysis [41]. The LR consists of eight butterfly-shaped subunits, each of which contain eight elongated, tubular protomers. Those protomers within the same LR subunit form a specialized domain, directly mediating the fusion between the inner and outer nuclear membranes. At the same time, two adjacent LR subunits also interact with each other forming another specialized domain with two conformations. This domain is believed to cushion the contacts among neighboring NPC particles.

The inner ring (IR) forms the NPC’s structural heart, making it one of the most structurally conserved regions in the entire complex. It is roughly symmetrical with two superimposed laterally offset rings: one located on the cytoplasmic side and the other on the nuclear side. The IR extends from the NE to the central channel, acting as a framework of membrane stabilization and the anchoring point for FG-Nups in the central channel [42–44]. Although the overall architecture of the inner ring is relatively conserved in organisms studied to-date, variation exists among the dimensions and the pattern of linkage with outer rings. One noticeable difference is the presence of an additional scaffold Nup called Nup155 in vertebrate cells, which forms pillar like structures connecting the inner rings to the outer rings [45]. It should be noted that this kind of connection is absent in yeast [46] and is only present on the nuclear side of the algal NPC [47]. Even though the functional reason for those pillars remains unclear, it is believed that they contribute to the size difference between the vertebrate and yeast NPCs.

The concept of the inner ring is a general idea based on the architecture of the NPC. Thus, there is no universal definition for the inner ring Nups. Some papers excluded Nup53 and Nup170 in yeast from the inner ring complex because they are classified as adaptor Nups [48], while others refer to the inner ring as the Nup170 subcomplex [49]. In some literature reviews, the vertebrate Nup62 complex combines with Nup98 forming an NPC substructure called symmetric FG-Nups [49], while others put the complex into the topographic map of the inner ring [42].

The two outer rings (also separately referred to as the cytoplasmic and nuclear rings) reside at the nucleoplasmic and cytoplasmic periphery of the NPC and sandwich the inner rings [45]. Those rings facilitate the smooth transition of the pore membrane into the inner and outer nuclear membrane [50]. In addition, these rings also serve as anchor points for asymmetric Nups such as the cytoplasmic Nups Nup358 and Nup214 that were discussed in the previous section, and nuclear basket Nups TPR and Nup153. Additionally, some studies suggest that the Nup84 complex, a yeast homolog to the vertebrate Nup107-160 Y-complex, the major components of the outer ring, combine with linkage Nups to act as the cytoplasmic docking sites for mRNA export factors [51,52].

Structurally speaking, the building block for the outer rings is the Y-complex mentioned in the previous section. The root of the Y shape contains membrane-binding motifs that contact the NE, while the entire subcomplex arranges in a lateral head-to-tail configuration, thereby forming the outer rings on both sides of the NPC [53]. Interestingly, even though the Y shaped building block of the outer ring is highly structurally conserved, its overall architecture varies from organism to organism. Two staggered rings, each with eight Y-complexes, were found on both cytoplasmic and nuclear rings in each vertebrate NPC, adding up to 32 copies of the Y-complexes in total [54,55]. On the other hand, only one octameric ring per side has been observed in yeast, meaning that yeast contains only half the number of Y-complexes when compared to vertebrate cells[50]. The algal NPC is somewhat intermediate, with double octameric rings on the nuclear side and a single ring on the cytoplasmic side [47].

Although most of the Nups in the NPC are organized into stable subcomplexes forming building blocks, such as the Y-complex, a small subset of scaffold Nups is not stably bound to any of the biochemically stable building blocks [56]. These Nups (often referred to as linker Nups or flexible connectors) contain long intrinsically disordered regions with short linear motifs (SLiMs), making them highly dynamic, exhibiting low-affinity interactions with other compounds in the NPC. Thus, they act as the dominant driving force to bridge interactions between and within different subcomplexes, including the outer and inner rings of the NPC and the channel Nups [44].

### Nuclear basket

The nuclear basket of the NPC is composed of the nuclear basket (BSK)-Nups, which consists of three members in vertebrates as Nup153 [57], TPR [58], and Nup50 (Figure 1a,b) [59]. The basket seems to be a flexible structure that reorganizes and rearranges during nuclear export, providing mRNPs with sizable molecular weight access to the central transporter [60,61]. The nuclear basket is also known to form a selective exclusion zone for heterochromatin [62] and certain large mRNPs [63]. This feature is believed to function as the NPC entrance for nucleocytoplasmic transportation. It is also believed to form chromatin boundaries that consist of actively transcribed chromatin regions [64,65]. On the structural level, the molecular architecture of the nuclear basket is largely nebulous. All three BSK-Nups contain contain intrinsically disordered regions that cannot fold spontaneously into stable three-dimensional globular structures. Thus, high-resolution crystal structures of the individual BSK-Nups are unobtainable at this moment due to technical limitations. The current models of nuclear basket structures rely on images obtained by various EM techniques combined with the biochemical properties and bioinformatics analysis of the BSK-Nups.

Nup153, named for its predicted molecular weight, is an FG-Nup in the nuclear basket that plays an essential role in RNA export [66] and protein import [67]. Nup153 antibodies block three major RNA export classes: snRNA, mRNA, and 5S rRNA, while the Importin α/β-mediated protein import was significantly reduced in the absence of Nup153. Recently, the importance of Nup153 on DNA double-strand break (DSB) repair was also documented [68,69]. 53BP1 is a DNA damage response (DDR) mediator and a tumor suppressor. The 53BP1 mediate the repairs for DSB with accumulation on damaged chromatin, promotion of DNA repair, and enhancement of DDR signaling. It has been reported that Nup153 promotes the 53BP1 import, thereby facilitating DSB repair. Furthermore, the knockdown of Nup153 reduced nuclear accumulation of 53BP1, delaying DSB repair. Besides the nuclear accumulation of 53BP1, SENP1, a SUMO protease, is also displaced in the absence of Nup153. Immunofluorescence microscopic images indicate that SENP1 partially mislocates to the cytoplasm instead of enriching the NE with Nup153 depletion. Such mislocation leads to reduced sumoylation of 53BP1, which is essential for an efficient accumulation of 53BP1 at sites of DSBs [70].

Structurally speaking, Nup153 contains three distinct regions, (1) a unique N-terminal region (1–649) contains a pore targeting interface, (2) A zinc finger region (650–880) contains four C2–C2 type zinc fingers that are most similar to zinc fingers found in Nup358, and (3) an FG rich C-terminal region (881–1475) contains 25 FG repeats (Figure 1b) [71]. The FG-enriched C-terminal region most probably plays a significant role in nucleocytoplasmic transportation due to its interactions with TAP and Importin-β1. The N-terminal region has NPC targeting activity interacting with TPR [72], the prominent architectural BSK-Nup, and the Nup107-160 complex in the central channel [73]. Thus, it is likely that this region facilitates the anchoring of Nup153 to the NPC superstructure. Nup153 is also essential in lamin fiber–NPC interaction to correctly distribute the NPC on NE. Studies have shown direct interaction between the N-terminal and C-terminal of Nup153 with Lamin A, Lamin B1, and Lamin B2 [74]. The interaction between Lamin B3, found only in amphibians and fish, was also documented [75]. Depletion or disruption of either Nup153^67^ or lamin genes [76] leads to the clustering of NPCs. In a more recent study utilizing Structured illumination microscopy (SIM), Nup153 depletion decreases the distance between lamin fiber and NPC and compacts the lamin meshworks [77]. No Nup in yeast shares the same overall domain composition as Nup153; Although specific functional and sequence features are shared with certain yeast BSK-Nups such as Nup1, Nup2, and Nup60 [71].

TPR is the BSK-Nups that constitutes the majority of the central scaffold of the nuclear basket. TPR, which stands for translocated promoter region, was initially described in the context of oncogenic fusions. Since then, various nuclear functions have been documented with the involvement of TPR, including nuclear transport [78], chromatin organization [62], regulation of transcription [79], and mitosis [80]. Sequences analysis and prediction suggest that TPR contains an N-terminal region with four coiled-coils and a C-terminal intrinsically disordered region (Figure 1c). However, due to technical limitations, the arrangement of the four coiled-coils remains unclear. One thing that needs to highlight here is that TPR only contains three FG repeats on its C-terminal intrinsically disordered region, which is the lowest among all FG-Nups. Although TPR could be catagrised as an FG-Nup, the functions of TPR are largely FG domain independent.

Little in the literature points to a specific or distinct role in the nuclear export for TPR. However, it has been reported that reduction in TPR function via an anti-TPR antibody or siRNA has been directly linked to disrupted Crm1-dependent protein export [58,81], suggesting a prominent role for TPR in macromolecule export. However, this deficiency remains as of yet unclear as to whether the disruption in Crm1-dependent protein export is due to a disruption in interaction with the karyopherin and the FG-region of TPR or some other, as of yet, uncharacterized function of TPR. The structural bioinformatics simulation data also suggested an interaction between TPR’s disordered C-terminal region and Crm1 [82].

At the same time, TAP, an mRNA export receptor, has been linked to TPR in some recent studies. Transcriptomic responses between TAP and TPR demonstrate a highly significant overlap of 72%, indicating that both proteins participate in the same pathway [83]. When altering TPR level, even modest knockdowns via RNA interference resulted in a significant increase in TAP-mediated mRNA export [84]. Further investigation indicated that the export of completely spliced mRNAs was not affected, and the difference was due to the upregulated export of unspliced mRNA. A similar result was consistently observed in three cell lines [85], indicating that TPR plays an essential role in the quality control of TAP-dependent mRNA transport. Surprisingly, a newly published paper showed that the TAP-TPR interaction fulfills the tRNA export [86]. Both TPR and TAP knockdown leads to tRNA accumulating in the nuclear of human lung cancer cells. Co-immunoprecipitation was only observed between tRNA and TAP, indicating TAP as the export receptor. Such results further expanded the functionality of TPR in RNA export beyond mRNA, suggesting an evolutionarily conserved TPR-TAP facilitated mechanism for exporting different types of RNA. Earlier structural studies showed possible interaction between the UBA (Ubiquitin Associated) domain of TAP and FG Nups [87,88]. However, the interaction between TPR and TAP has not been detected so far. Pores observed by STED superresolution microscopy showed no adjacent TAP to TPR [89]. Their interactions could not be detected through immunoprecipitation as well [85]. At the time of the writing of this manuscript, there is no direct evidence that TPR has a role in classical NLS-dependent import; however, despite a lack of characterized involvement in nuclear import TPR has been reported to bind the import receptor Importin-β1 [90].

Compared to the abundant amount of research on TPR and Nup153, studies on Nup50 are somewhat limited. Most of the research on Nup50 combines it with Nup153, forming the Nup153-Nup50 protein interface (Figure 1b). Associations have been suggested between the Nup153-Nup50 protein interface with 53BP1-mediated DSB repair [91] and nuclear import [92]. Nup50 is largely disordered with two ordered terminal regions (Figure 1c). The N-terminal region has characterized Improtin-α binding segments and a Nup153 binding site, while the C-terminal region contains a RanBD1 (Ran Binding Domain 1) domain, allowing Nup50 to interact with Ran GTPase. Interestingly, some evidence has suggested a transport-independent and Nup153-independent role for Nup50 in chromatin biology that occurs away from the NPC [93].

### Nucleocytoplasmic transport models

NUPs within the NPC form the selectivity barrier that prevents the passive diffusion of larger molecules, instead requiring mediated transport by a karyopherin, also referred to as a transport receptor, belonging to the ~20 member large importin-β superfamily [13]. This super family of karyopherins typically transport cargo in a unidirectional fashion allowing for the distinct classification of specific karyopherins as importins or exportins dependent upon the direction of translocation, although it must be noted that some karyopherins have been observed to function in a bidirectional fashion [94]. To facilitate transport, karyopherins interact with proteins or intermediary proteins, such as importin-α, to facilitate transport through the NPC. This is accomplished through a variety of multivalent interactions between the transport receptor and the hydrophobic FG-Nups within the central channel of the NPC [95]. While much remains unknown about the gating mechanisms of the NPC, the interactions, while unique to each individual karyopherin, remain largely similar in that a series of hydrophobic grooves on the surface of HEAT-repeats interact with the hydrophobic FG-Nups in a balanced fashion [96], where the interactions are sufficiently strong to ensure association, and therefore export, yet sufficiently weak and transient enough to promote fast translocation through the NPC [23,95,97,98].

Multiple nucleocytoplasmic models have been proposed to help understand the basic nuclear transport mechanism for small and large transiting molecules, including (1) The Brownian/virtual gate/polymer brushes model, (2) the selective phase/hydrogel model, (3) The reduction of dimensionality (ROD) model, and finally (4) the two-gate/forest model.

The Brownian/virtual gate/polymer brushes model assumes that the non-interacting FG-Nups provide an energy/entropy barrier; Such a barrier strictly hinders randomly moving inert molecules. The proposal is based on the net positive charged FG-Nups, like ‘polymer brushes’ that repel charged molecules in their surroundings [1,99]. The model experimentally demonstrated the entropically repulsive or thermally exclusive behavior of Nup153 [100,101]. In contrast, the selective phase/hydrogel model argues that the FG repeats of FG-Nups interact hydrophobically and predominantly between phenylalanine residues to form a cohesive meshwork or hydrogel. The meshwork aligned across the entire NPC channel, providing the barrier that blocks all unwanted large molecules from passing. Numerous holes in between the connections allow the passage of small molecules. The binding between cargo-NTRs and FG domains dissolve through the hydrogel, allowing the cargo-NTRs to pass through the NPCs [102,103]. Alternatively, the reduction of dimensionality (ROD) model suggests that the collapsed central channel FG repeats coat the central walls in parallel, providing layers for the cargo-NTRs to travel through. Such movement is much like work in 2-dimensional (2-D) rather than 3D Brownian movement. Finally, the 2-gate/forest model is the newest model being proposed: the non-cohesive FG repeats in the peripheral sides of the channel act as the repulsive gate, which the cohesive FG repeats in the interior of the central channel function as the selective gate [104,105].

## Mechanisms of mRNA nuclear export

The export of RNA occurs in a predominately unidirectional manner with few exceptions[13] via a variety of transport receptors; most prominently, TAP-p15 and Crm1. While both of these transport receptors function to export mRNA, they differ wildly. Most fundamentally TAP-p15 and Crm1 differ in that TAP and its cofactor p15 are not members of the karyopherin family and facilitate nuclear export in a Ran independent manner [87,106]. Conversely Crm1, also called Exportin-1, belongs to the karyopherin family and functions in a Ran dependent manner [107–109]. In addition, there is a significant difference in the cargoes transported by Crm1 and TAP and its cofactor p15 (TAP-p15). TAP-p15, also called NXF1 and Nxt1 respectively, exports the vast majority of mRNA in a RAN independent manner often referred to as bulk mRNA export [33,110,111]. Whereas Crm1 is the major workhorse of macromolecular nuclear export including hundreds of nuclear export signal (NES) containing cargo proteins [112–114], as well as RNAs that are exported with the assistance of adapter proteins: these include a small subset of mRNAs, rRNA, and snRNAs [115,116]. Notably, Crm1 only exports a small portion of cellular mRNA. This pathway is commonly referred to as specific export and includes several protooncogenes and cytokines that contain AU-rich elements (ARE) in their 3’ untranslated regions [109,117–120]. This pathway makes use of three adaptor proteins to facilitate export of mRNA: RNA-binding protein human antigen R (HuR), leucine-rich pentatricopeptide repeat protein (LRPPRC), and nuclear export factor 3 (Nxf3). HuR associates with ARE and then associates with APRIL, pp32, and Crm1 to facilitate export [121,122]. Nxf3 is intriguing as it belongs to the Nxf family but lacks the c-terminal export signal enabling the export of RNA and therefore functions in a Crm1 dependent manner [123,124]. LRPPRC functions by associating with eukaryotic translation initiation factor eIF4E and Crm1 to facilitate export. RNAs exported via this pathway contain an ~50-nucleotide eIF4E sensitive element (4ESE) found in both the 3’ UTR and the 7-methylguanisine cap. The 4ESE elements in both the 5’ cap and the 3’ UTR interact simultaneously with LRPPRC, which in turn interacts with Crm1 to facilitate export [109,125].

Bulk export of mRNA within eukaryotic cells occurs through several stages: Trafficking within the nucleus following transcription, docking at the nuclear basket of the NPC, translocation through the NPC, and release into the cytoplasm from the cytoplasmic fibrils on the cytoplasmic face of the NPC [120,126–129]. During and immediately following transcription pre-mRNA is processed for nuclear export, this processing includes splicing, the addition of a poly-adenylated tail, and the addition of a 5’-7-methylguanosine cap [130–134]. During and following processing, mRNA recruits a series of proteins to form mRNA:protein complexes (mRNPs) including a wide variety of proteins, notably the transcription export complex (TREX), consisting of UAP56, REF/a Aly, CIP29, and the THO multi-subunit complex [135]. A nuclear transport receptor, TAP and it’s co-factor p-15 are also recruited and together, TREX and TAP-p15 are the factors primarily facilitating intranuclear transport leading to docking in the nuclear basket of the NPC [120,136–142].

Docking at the NPC and export of mRNPs are directly facilitated by the interaction between the transport receptor and the FG-Nups found in the NPC. The interaction between transport receptor and FG-Nups have been hypothesized to function as the initial interaction point allowing for docking of mRNPs at the entrance of the NPC as well as a critical interaction required for export through the NPC via a facilitated diffusion mechanism [120,126,143–146]. Notably, the interaction between FG-Nups and transport receptors does not appear to confer directionality upon the cargo, as is evidenced by similarity of interaction between import and export interactions between transport receptors and FG-Nups [126,146,147]. Upon passing through the FG-rich central channel of the NPC, mRNPs then dissociate from cytoplasmic fibril Nup214 via essential mRNP export factors Gle1, IP6, and DDX [35,129,148–152] and difuse into the cytoplasm.

During dissociation, Nup358 plays a key role in the release and recycling of transport receptors and co-factors. As has been previously mentioned, Nup358 contains binding domains for TAP-p15 dimers as well as RanGAP, RanGTP and RanGDP. TAP-p15 dimers associate with Nup358 and are prevented from further diffusion into the cytoplasm [153], while Ran dependent exportins dissociate via hydrolysis of RanGTP to RanGDP via RanGAP [154]. RanGDP is then recycled via nuclear transport factor 2 (NTF2), which associates with RanGDP and facilitates import into the nucleoplasm where the RanGEF exchange factor RCC1 enables nucleotide exchange to RanGTP, enabling it to once again facilitate Ran dependent export [154,155].

In a simplified form, this export model has five discrete steps. First, the mRNP is formed along with all requisite cofactors and associates with transport receptors. Next, the transport receptor interacts with FG-Nups to facilitate docking and initiate export. Upon successful docking and initiation, the mRNP then moves through the central channel of the NPC via interactions between the FG-Nups and the hydrophobic regions on the transport receptor. After successfully passing through the central channel, the mRNP then releases from the cytoplasmic fibrils and diffuses into the cytoplasm.

## Basket nups and their impact on nuclear export of mRNA

The widely accepted model of mRNA export described in the previous section is predicated upon the understanding that transport receptors directly interact with FG-Nups to facilitate docking, a phenomenon that has been widely observed. However, the role of the individual basket Nups upon the docking and subsequent export process have been largely overlooked. Recent publications have highlighted critical functions for basket Nups in the docking and subsequent export of mRNA through the NPC [73,114].

Using a combination of Single-Point Edge-Excitation sub-Diffraction (SPEED) microscopy and Auxin Inducible Degron (AID) technology, a technology that enables the rapid and highly specific degradation of target Nups, a pair of recent publications have made great strides in unraveling the relationship between basket Nups and the docking and export of mRNA [83,129]. The utilization of these two techniques in concert has revealed that basket Nups play a much larger role in nuclear docking and export than previously thought. The widely accepted thinking on docking of mRNA at the NPC is that a direct interaction between FG-Nups and the transport receptor is responsible. However, recent evidence suggests both that specific FG-Nups in the nuclear basket have specific roles in the docking and export efficiency of mRNA as well as that interactions between the TPR and the mRNP are critical for successful nuclear export. Notably, the observations that blocking Nup153 or TPR with anti-Nup153 or anti-TPR antibodies were found to inhibit mRNP transport from the nucleus to the cytoplasm [66,156,157]. In support of these observations, it was also reported that anti-Nup153 and anti-TPR antibodies resulted in nuclear accumulation of mRNA in eukaryotes [83,158].

In their 2020 manuscript, Aksenova and colleagues demonstrated the viability of evaluating the impact of the absence of individual basket Nups using AID strategies. Prior to the utilization of this strategy, researchers remained largely unable to explore the loss of individual basket Nups. This is due to limitations of other inhibition strategies. Nup153, Nup50 and TPR have been observed to have long half-lives of ~20–30 hours in tissue-culture cells in interphase [156]. However, RNAi-mediated Basket Nup degradation strategies require more than 72 hours and multiple rounds of cell division [72,159,160]. This is problematic in two respects, first the degradation required is longer than the half-life of the Nup within the nuclear basket. Second, the NPC is largely stable during interphase [74,161]. During post-mitotic assembly the absence of specific Nups can impact the overall structure and stability of the pore, disallowing it from forming a complete structure and thereby failing to provide researchers with an accurate picture of the impact of that specific Nup on the export behavior of mRNA. By utilizing AID strategies, Aksenova and colleagues demonstrated an ability to degrade a specific Basket Nup in ~1 hour. Further, they demonstrated that the loss of individual Basket Nups did not grossly impair either the composition or architecture of the NPC[83].

In addition to demonstrating the utility of the AID technology, Aksenova and colleagues provided support for unique roles of Basket Nups in mRNA export. Specifically, the degradation of the low-FG-repeat containing Nup TPR was found to cause significant changes in transcriptomic profiles of mRNA in a fashion similar to that observed when TAP or GANP, a subunit of TREX-2, was degraded. Further, the degradation of TPR resulted in a disruption of association between GANP, PCID2, and ENY2, all TREX-2 subunits, with the NPC, indicating an important role for TPR in gene expression of mRNAs that utilize the pathways mediated by TAP-p15 and/or TREX-2^83^. Of particular interest is the finding that GANP is not only required for recruitment of TREX-2 complex subunits [162], but appears to be the primary factor in docking at the nuclear basket via interaction with TPR. The transport receptor TAP was likewise evaluated and found no evidence that it plays a role in docking to TPR [83].

**Figure 2.**
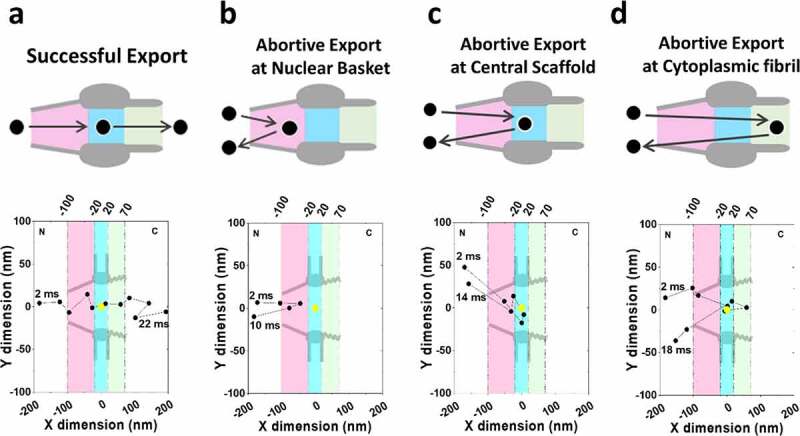
**Models of successful and abortive nuclear export of mRNPs tracked via high-speed single-molecule SPEED microscopy**. (a) A model of successful export coupled with a typical successful export trajectory. (b) A model of abortive export at the nuclear basket coupled with a typical nuclear basket abortive export trajectory. (c) A model of abortive export at the central scaffold coupled with a typical central scaffold abortive export trajectory. (d) A model of abortive export at the cytoplasmic fibril coupled with a typical cytoplasmic fibril abortive export trajectory. partial figure reprinted with permission. originally published in proceedings of the national academy of sciences.[129]

In another study focused on the dynamics of mRNA export in the absence of targeted Basket Nups, Li and colleagues employed AID strategies to observe the alterations in export behavior or mRNA via SPEED microscopy [129]. SPEED microscopy is a super-resolution technique permitting a spatial localization precision during *in vivo* imaging of approximately 10 nm and a temporal resolution up to 0.4 ms that has been employed to great effect to track macromolecules as they move through the NPC or primary cilium of the cell [144,163–167]. In addition to deriving dynamic information regarding the docking and export behavior of macromolecules, SPEED microscopy makes effective use of a 2D-to-3D transformation algorithm. This algorithm takes localizations captured within a rotationally symmetrical structure, such as the NPC or primary cilium, and is able to derive three-dimensional distributions of the localizations within the structure. Thereby providing virtual 3D single-molecule microscopy data derived from an *in vivo* 2D dataset [144,163,164,168–171]. This level of spatial and temporal resolution in both 2D and 3D provides researchers with significant information related to the docking behavior, export pathways through the NPC, and export efficiency of the mRNA where efficiency. This information can be further divided by evaluating export events in specific sub-regions of the NPC. Specifically, export events are classified into four different categories: Successful (Figure 2a), abortive export at nuclear basket (Figure 2b), abortive export at central scaffold (Figure 2c), and abortive export at cytoplasmic fibril (Figure 2d). This highlights that not all mRNP encounters with the NPC are productive resulting in export. In fact, many mRNPs will fail to export and then be re-imported into the nucleoplasm. This allows researchers to evaluate the regional exportive failure due to the absence of a specific basket Nup as well as the total export efficiency, which is defined as the number of successful export events divided by the total number of successful and abortive events [129].

The combination of AID and SPEED microscopy allowed for several novel observations. First, by sequentially degrading basket Nups and imaging with SPEED microscopy, the stoichiometric ratio of Basket Nups was found to be 1:1:1. Secondly, individual Basket Nups play distinct roles in the export of mRNAs. Thirdly, a minimum copy number of Basket Nups required to provide function was identified. Lastly, the loss of individual Nups from the nuclear basket were evaluated for their impact on the three-dimensional export routes of mRNA as it moves through the NPC. Together this information, in tandem with the information derived by Aksenova and colleagues paints an interesting picture of the roles of Basket Nups on the export of mRNA.

**Figure 3.**
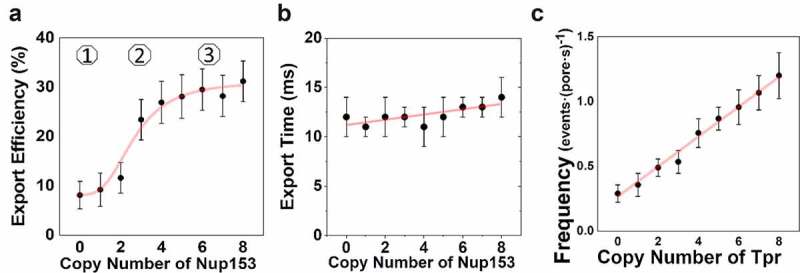
**Correlation between copy number of Nup153 and its impact on mRNP export dynamics**. (a) the copy number of Nup153 present within the NPC directly impacts the efficiency of export. numbers denote the three phases between the nuclear export efficiency of mRNAs and the copy number of Nup153. (b) the copy number of Nup153 unlikely impact export time of mRNPs. (c) correlation between docking frequency and the copy number of Tpr. partial figure reprinted with permission. originally published in proceedings of the national academy of sciences.[129]

As was mentioned previously, the widely accepted model of nuclear export of mRNA relies upon the interaction between transport receptor and FG-Nup to facilitate docking at the NPC. Interestingly, the Basket Nup TPR, a low-FG-repeat-Nup, appears to be of critical one in nuclear docking. As was demonstrated by Aksenova and colleagues, the absence of TPR results in a significantly altered mRNA expression profile akin to those observed by the complete knockout of TAP or the structural subunit of TREX-2, GANP. While it was observed that TREX-2 is tethered to the NPC via TPR, it remained unclear if the differential transcriptomic expression observed in the absence of TPR and/or GANP is the result of an export deficiency or a processing deficiency. Evaluation of the role of TPR in mRNA export using dynamic super-resolution data indicated that degradation of TPR resulted in a four-fold decrease in docking frequency of the mCherry-tagged mRNA in the ΔTPR condition. Further, when the copy number of TPR was evaluated for correlation between docking frequency, it was observed that the docking frequency increases in a direct correlation between copy number of TPR (Figure 3c). However, the loss of TPR had little direct impact on the percentage of successful export when compared to the wild type. The fourfold decrease in docking frequency in the absence of TPR suggests that this interaction plays a significant, and perhaps even critical, role in the docking of mRNPs at the NPC. It is therefore probable that the differential transcriptomic profile observed by Aksenova and colleagues is most probably caused by the disrupted interaction between TPR and TREX-2; suggesting that this relationship may have a role in the final processing of mRNA, an idea supported by the reported observations that knockdown of TPR results in an increased export of unspliced mRNA [84].

To further characterize the nature of the interaction between TPR and mRNPs, it must be examined in greater detail whether the interaction between an mRNP and TPR leading to docking is due to an interaction between TREX-2 and TPR, or perhaps both TREX-2 and TAP-p15 interacting with TPR. As only TAP was evaluated for its role in docking via TPR, it must be considered that the TAP-p15 dimer may have an impact on the docking at the nuclear basket, particularly when functioning in concert with TREX-2. In addition, the question must also be answered whether the dynamics of plasmid derived mRNA interacts in a manner distinct from endogenous mRNA. To further probe the interaction, *in vivo* SPEED export studies of endogenous TPR-GANP dependent mRNAs should be evaluated for export efficiency to determine if the export of plasmid derived mRNA differs from endogenous mRNA.

Subsequent degradation of the FG-Nups Nup50 and Nup153 showed virtually no impact upon the docking frequency of mRNAs at the NPC. This suggests a fundamental shift in thinking as the low-FG-repeat containing Nup appears to have the single largest impact upon docking events at the NPC. Interestingly, the lone deletion of Nup50 appears to have little to no impact upon either the docking frequency or the export efficiency of mRNAs while Nup153 had a significant impact on the export efficiency. Degradation of Nup153 led to an approximately fourfold decrease in export efficiency from ~31% in wild type to ~8% in ΔNup50-ΔNup153. In light of the observation that Nup50 anchors to Nup153, degradation of Nup153 effectively removed Nup50 from the nuclear basket but does not degrade Nup50 from the cell. Due to the lack of impact caused by the degradation of Nup50, the primary basket Nup involved in export efficiency is Nup153.

Of particular interest was the observation that a minimum copy number of Nup153 present within the nuclear basket enables near full recovery of export function (Figure 3a,2b). The relationship between export efficiency and copy number of Nup153 appears to be sigmoidal in nature with a sudden increase in efficiency observed when copy number increases to 4 with only a minor increase in efficiency observed as the full complement of Nup153 copies is present in the NPC. This correlation only held true for export efficiency as export time remained unaffected by the copy number of Nup153, suggesting that Nup153 has an important role in initiation of export through the NPC, but is only impactful during the beginning stages of the process. Interestingly, TPR was found to increase docking frequency in a direct correlation to the number of copies found in the NPC (Figure 3c). Together, this data suggests that TPR functions to facilitate docking to the nuclear basket while Nup153 functions to initiate export through the NPC.

**Figure 4.**
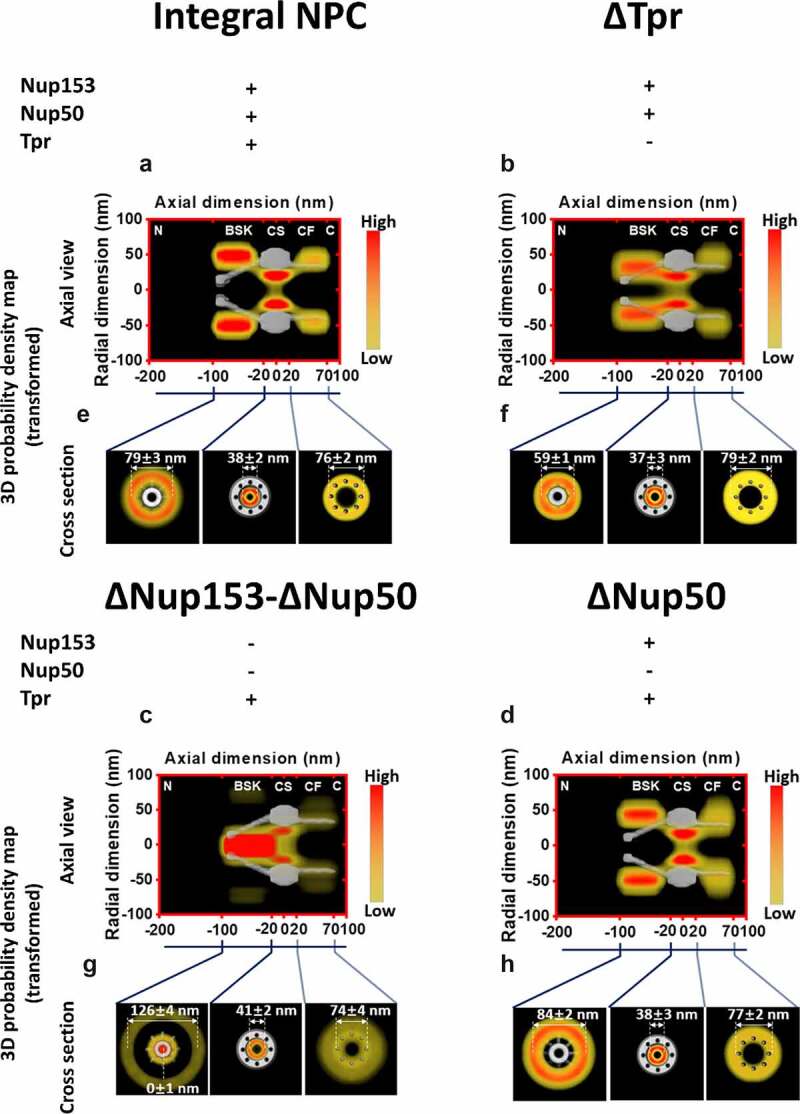
**Three-Dimensional export routes of mRNPs**. **(A-D)** An axial view of the 3D probability density maps for mRNPs under different conditions. Shown here is the entirety of the NPC, including nuclear basket, central channel, and cytoplasmic fibrils. Red indicates high probability localizations while yellow indicates a low probability localization. (e-h) A radial view of 3D probability density maps for mRNPs under different conditions. shown here are subregions of the NPC including nuclear basket, central channel, and cytoplasmic fibrils. partial figure reprinted with permission. originally published in proceedings of the national academy of sciences.[129]

The sequential deletion of basket Nups also has been indicated to impact the 3D export routes of mRNA. In the absence of TPR, it is shown that export routes become less well defined, and probability density appears to follow the localization of Nup153 in the nuclear basket (Figure 4b,f). The most dramatic change is observed when Nup153 is degraded resulting in ΔNup50-ΔNup153. Here we see a shift from the periphery of the nuclear basket observed in the integral NPC (Figure 4a,e) to a localization near 0 in the radial dimension. This localization and shift is reasonable when one considers that TPR appears to promote docking and Nup153 appears to initiate export through the NPC. In the absence of the high-copy-FG-Nups in the nuclear baskets, the transport receptors congregate near the center of the nuclear basket until interacting with FG-nups in the central channel (Figure 4c,g). This highlights the distinct and separate roles between the discrete Basket Nups.

Lastly, the highly targeted and specific degradation of Nups facilitated by AID further allowed Li and colleagues to evaluate the stoichiometric composition of the Nups that form the nuclear basket. Using a combination of super-resolution microscopy techniques and AID strategies, it was discovered that Nup50, Nup153, and TPR exist within the nuclear basket in a 1:1:1 stoichiometry. Further, it was shown that degradation of Nup153 caused the depletion of Nup50 from the pore, but not the cell. This further confirms that Nup50 is anchored to Nup153 within the nuclear pore complex. Of interest here is the implication that this stoichiometric composition has upon the selectively permeable barrier in the NPC. All three basket Nups, Nup50, Nup153, and TPR, contain FG repeats, with TPR having the fewest at 3 copies, Nup50 with the next fewest at 5 copies, and Nup153 with the most at 25 copies. Due to a balanced composition of Nups, the contribution of each basket Nup to nuclear export can be evaluated in an equal manner. With this in mind, the ΔNup153-ΔNup50 cells exhibited a nearly four-fold decrease in successful export events while the ΔNup50 cell lines were not significantly different from the wild-type. This suggests that the Nup153-Nup50 complex, or more probably Nup153 alone, is the critical component to the initiation of export through the NPC. This is most probably due to the significantly higher number of FG-repeats found in Nup153 when compared to Nup50 and TPR; five-fold and eight-fold respectively.

Similarly, as TPR was found to have no significant impact on export efficiency but was demonstrated to have the single largest impact upon docking frequency, it must be concluded that TPR functions primarily in facilitating docking. This function is likely in a manner separate and distinct from the FG-Nup interaction between transport receptors that has been previously put forth, as TPR has the fewest FG-repeats of the basket Nups and has a near identical number to Nup50; 3 copies and 5 copies, respectively. Further, it is unlikely that the discrepancy is due to the location of TPR within the nuclear basket. The close proximity of TPR to Nup153 and Nup50 would likely compensate for the loss of TPR if the interaction between FG-nup and transport receptor were the primary driving force behind docking.

**Figure 5.**
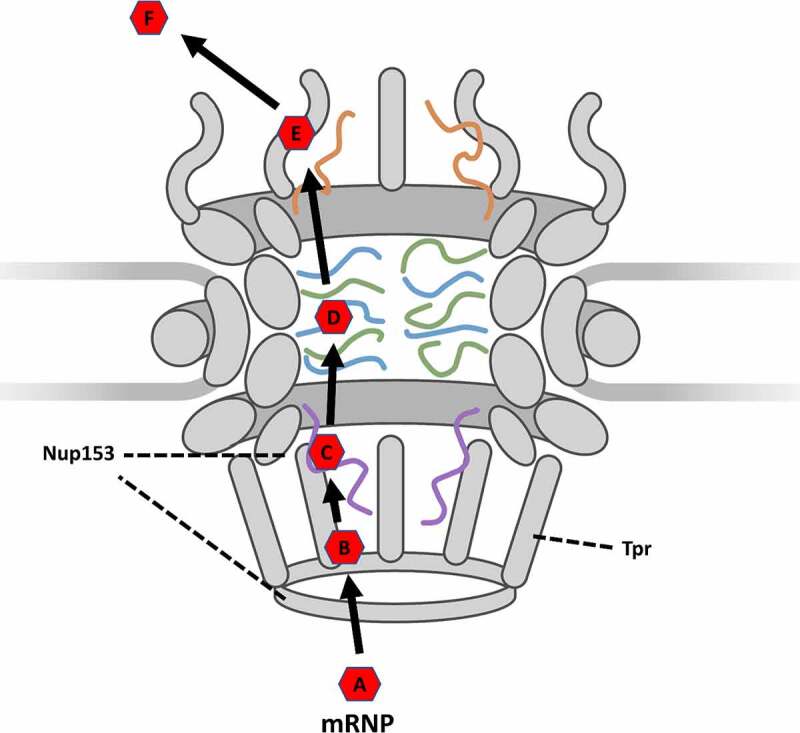
**Updated simplified model of mRNP export as it moves through the NPC**. **(A) formation**: the formation of an mRNP including nuclear transport receptor and other co-factors. **(B) docking**: Docking at the nuclear basket via interaction with TPR. **(C) initiation**: Initiation of export by interactions between nuclear transport receptor and Nup153. **(D) transport**: Movement through the central channel facilitated by interactions between transport receptor and FG-Nups. **(E) Release**: Release from the cytoplasmic fibrils. **(F) diffusion**: Translocation from the NPC to the ribosome.

These observations together suggest that the widely accepted model of mRNA export may need to be updated. Rather than simply an interaction between FG-Nups and the transport receptor being responsible for docking and initiation of export, it is more probably docking facilitated by the low-FG-repeat-Nup TPR interacting with mRNA co-factors such as TREX-2 and then initiating export through the NPC by interactions between Nup153 and the transport receptor. The updated model then would indicate that following transcription and recruitment of co-factors, a fully formed mRNP (Figure 5a) would translocate to the nuclear basket where TREX-2 interacts with TPR in a docking step (Figure 5b). Transport receptors would then be positioned to interact with Nup153, which positions the mRNP near the inner ring of the central channel in an initiation step (Figure 5c). The mRNP would then proceed following the accepted model where hydrophobic domains on the exterior of the transport receptor interact with FG-Nups in the central channel in a transport step (Figure 5d) to move through the NPC until releasing from the cytoplasmic fibrils in a release step (Figure 5e). The mRNP would then proceed to translocate to the ribosome in a diffusion step (Figure 5f). This updated model thereby demonstrates the unique contributions and roles that basket Nups have upon nuclear export and provides researchers with a more detailed model of the discreet steps required for nuclear export of an mRNP.

## Future Perspective

### What are the individual impacts of basket nups on Crm1 mediated mRNA export?

While the recognition of the specific roles played by basket Nups in the export of mRNA has been a powerful and impactful one, there still remains much unknown regarding the specific roles of individual proteins in export. One particular area of interest will be the impact of nuclear basket Nups on the export of mRNAs mediated by Crm1. The firefly luciferase mRNA utilized to determine the export impact of Nup153, Nup50, and TPR is mediated via TAP-p15; which, as was indicated earlier, is both a part of the bulk export pathway and reliant upon TPR for proper mRNA processing and docking. To further evaluate the individual impact of these Nups on the export of mRNA, this methodology should be employed for Crm1 mediated specific mRNA export. The aim of this experiment will be to determine if the Basket Nups behave in a manner similar to those observed interacting with bulk mRNA export. Of particular interest will be the function of TPR in the specific mRNA export pathway as TPR functions to interact with and tether TREX-2 to the nuclear basket, thereby facilitating docking and probable processing of the cargo mRNA, in the bulk export pathway. Crm1 mediated specific mRNA export does not involve TREX-2 in any accepted models. Furthermore, TPR has been reported to have a significant impact upon Crm1-dependent protein export. It would therefore be of particular interest to evaluate if TPR has a similar impact upon docking frequency of mRNA to that observed in the bulk mRNA TAP-p15 mediated pathway. Further, as has been demonstrated, Nup153 is, among the basket Nups, the primary agent responsible for initiating export with Nup50 having only a negligible role. Given the observation that Nup50 and Nup153 have differential impacts upon the export of mRNA, is it then possible that Nup50 may play a larger role in specific mRNA export? If so, this would suggest that the structure of the nuclear basket is optimized for successful export of multiple pathways without interfering unduly with one another.

## How do other FG-nups and structural nups individually contribute to the export of mRNA?

The combination of SPEED microscopy and AID technology have been utilized to interrogate roles of individual nuclear basket Nups in NPC docking and the export of mRNA[129]. The study not only found that individual nuclear basket Nups have specific roles in mRNA export through NPC, but also the unprecedented relationship between TPR and NPC docking of mRNA. The AID facilitates the rapid and controllable degradation of Nups, while SPEED microscopy makes it possible to track the dynamics of mRNA export at super-spatiotemporal resolution in live cells. Given the advantage of AID and SPEED microscopy, the combination of the two approaches could be extended to interrogate the roles of other FG-Nups in nuclear export of mRNA.

FG-Nups were reported to play pivotal roles in mRNA export though the NPC via interacting with mRNA export receptors [33,172–176]. For central scaffold FG-Nups, the Nup62 subcomplex and Nup98 were found to participate in mRNA export [175,177–179]. The Nup62 subcomplex is a central channel Nup heterotrimer consisting of Nup62, Nup58 and Nup54. Nup62, Nup58 and Nup54 harbor numerous FG repeats (15, 14, and 9, respectively), which heavily contribute to the selectively permeable barrier of the NPC as well as the nuclear transport of macromolecules [48,56,180]. Another central channel Nup, Nup98 has been reported as a major component of the selectively permeable barrier of the NPC [181], and contains Glycine–leucine–FG (GLFG) which play a critical role in cohesiveness of permeability barrier of the NPC [182,183]. Thus, the Nup62 subcomplex and Nup98 may function in mRNA export by providing FG-repeats as anchoring sites for mRNA export receptors. Further, while not a part of the nuclear basket itself, it is possible that Nup98 has significant overlap with basket Nups and should be further interrogated for its role in docking and export.

Regarding cytoplasmic FG-Nups, the presence of Nup358 and Nup214 were reported to be essential in mRNA export [153,184] and of particular importance during the late stages of mRNA export [37,185]. Specifically, Nup358 functions as a docking site for TAP-p15 and was reported as a facilitator rather than an indispensable component of mRNA export[153]. Nup214 is involved in nuclear mRNA export by interacting with DEAD-box ATPase (Dbp5/DDX19) that remodels mRNP at the cytoplasmic side of the NPC [185]. In addition to Nup358 and Nup214, hCG1 (Nup42) is reported to bind to Gle1/GLE1 which activate Dbp5/DDX19 and is suggested to have critical roles in the final steps of mRNA export [35,150,186]. As a central channel FG-Nup, Nup98 also diffuses into cytoplasmic filament and binds to RAE1 which is an mRNA export factor required for the localization of Gle1/GLE1 [187,188].

However, the specific functions of these FG-Nups remains unclear and several core questions are remain unanswered; specifically, 1) Do FG-Nups in the same sub-region of NPC have equal functions in mRNA export? 2) In the absence of specific FG-Nups, is it possible for function to be retained via compensation by other FG-Nups? 3) What is the relationship of mRNA export kinetics with the copy number of specific FG-Nups? These questions could be comprehensively interrogated via a combination of AID strategies and SPEED microscopy. Specifically, AID allows for highly controllable removal of specific FG-Nups within NPCs while SPEED microscopy is applied to track mRNPs export through NPC of live cell in absence of specific Nup. These two technologies in concert will enable researchers to detect the dynamics of mRNPs as they export through incomplete NPCs.

## Alternative approaches for live-cell mRNA tagging for subsequent single-molecule super-resolution imaging

**Figure 6.**
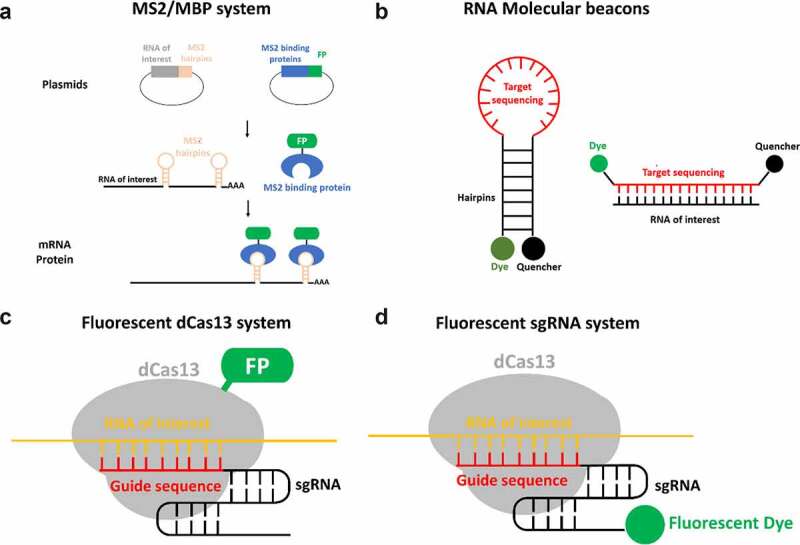
**Alternative approaches of live-cell RNA tagging for subsequent single-molecule imaging super-resolution imaging**. (a) The MS2/MBP system requires the construction of two plasmids. One construct encodes the RNA of interest with MS2 hairpin repeats. Another construct encodes the MS2 binding protein (MBP) fused with a fluorescence protein such as GFP. By transfection, both plasmids are transcribed and the MBP-FP are translated. The exogenous RNA of interest is recognized and bound by the fluorescent MBP. (b) The molecular beacon structure is a stem-loop probe. The stem-loop structure brings the fluorescent dye close to the quencher molecule. This quencher absorbs the energy emitted by the dye, reducing fluorescent background noise. When the loop containing the targeting sequence hybridizes with the RNA of interest, the hairpin dissociates. This separation moves the fluorescent dye out of range of the quencher, allowing emission of detectable fluorescence. (c) The assembly of fluorescent protein fused dCas13 protein (dCas13-FP) and sgRNA is directed by guide sequence of sgRNA to bind to RNA of interest and label target RNA with dCas13-FP. (d) the assembly of dCas13 and fluorescent sgRNA (F-sgRNA) binds to the RNA of interest and labels the target RNA with sgRNA conjugated with fluorescent dye.

In addition to AID and SPEED microscopy, Li and colleagues utilized the MS2/MCP tagging system to label the exogenous mRNA in live cells [129]. The MS2/MCP tagging system is based on the coat protein of the MS2 bacteriophage, which contains an RNA-binding site for RNA hairpin structures [189]. The MS2 coat protein (MCP) does not bind to the endogenous RNA of human cells, since the RNA hairpin structures do not exist in mammalian nucleic acids. In the MS2/MCP tagging system (Figure 6a), two constructs are depicted. One plasmid construct contains the mRNA of interest tagged with multiple MS2 hairpins and the other plasmid express the protein MCP fused with a fluorescent protein (FP). These plasmids are introduced in tandem to a cell [190]. The signal of the mRNA of interest is then significantly amplified when compared to free MCP-FP due to the presence of multiple MS2 hairpins, each of which can recruit an MCP-FP. Through this method, researchers are able to differentiate between free MCP-FP and MCP-FP associating with the mRNA of interest. In this manner the MS2 system has been employed in live-cell tracing mRNA in several organisms including yeast, mammalian, and insect [191–196].

The MS2/MCP tagging system has an important advantage for live cell tracking of mRNA. The MS2 hairpin repeats should not impede normal functions of the RNA. Theoretically, because MS2 hairpins are generally added to the distal end of the 3′UTR, and MCP binds MS2 hairpins exclusively, the addition of the binding of MCP to the RNA should not interfere with standard function. Despite this significant benefit, this approach has several limitations. 1) The extra efforts are required to construct a new plasmid for each RNA of interest. 2) Given the difficulty of inserting the MS2 hairpin repeat sequence into endogenous DNA, this approach is generally used to label exogenous RNA of interest. 3) The transcription of long sequences of palindromic DNA repeats is unstable and lead to slips in the DNA polymerase, which may result in loss of these hairpins [197]. 4) Unbound MS2-FP molecules contribute high levels of background noise and lower signal-to-noise ratios [198]. 5) This system is not amenable to multiplexing, since two different RNA-MS2 cannot be distinguished by MCP-FP [190]. In light of these limitations, other live-cell RNA labeling could be alternative approaches to compensate the drawbacks of MS2/MCP tagging system.

A relatively recent advancement in RNA labeling technology is RNA molecular beacons. This technique uses a similar mechanism as FISH, where fluorescently tagged oligos bind complementary to RNA of interest, but couple a quencher to the fluorophore in its unbound state [199]. In their unbound state, molecular beacons form a stem-loop which places the quencher and fluorophore in close proximity. In this conformation a quencher absorbs the energy emitted by the fluorophore and converts light to heat, dramatically reducing the background signal of unbound probes (Figure 6b) [200]. When binding to the target sequence separates the attached fluorophore and quencher the fluorophore is no longer being inhibited by the quencher, thereby allowing the fluorophore to emit fluorescence. Obviously, RNA molecular beacons have two advantages: 1) unbound molecular beacons wouldn’t cause high levels of background fluorescent as the fluorophore is reduced by quencher; 2) molecular beacons are amenable to multiplexing [201]. However, there is a potential risk associated with this technique. Specifically, there is a chance that the complementary covering of target RNA may disrupt normal processes such as trafficking or loading into an RNP complex [190].

CRISPR/Cas-based technologies have been widely employed in genome editing and genetic engineering [202]. Furthermore, this approach was expanded to track RNA/DNA with fluorescently tagged Cas protein in the live cells [203]. In the Cas system, dCas13 (catalytically inactive Cas13) has recently been utilized to track the dynamics of RNA in living cells [204]. When dCas13 is genetically fused with a fluorescent protein (FP) and assembled with an sgRNA (single guide RNA) that contains a specific target sequence that allow for the fluorescently labeling of RNA of interest in live cells (Figure 6c). This approach has two key advantages: 1) the ability to label the endogenous RNA in a live cell; 2) the low level of background noise in the cytoplasm due to accumulation of unused dCas protein [205,206]. This approach also has several shortcomings. 1) This approach requires a significant effort to optimize the guide sequence of sgRNA [205]. 2) The dCas13/sgRNA complex binding to the target RNA may disrupt other RNA binding proteins. 3) The high level of background noise due to the nuclear accumulation of unused dCas13-FP. 4) This system isn’t amenable to multiplexing because dCas13 assembles indiscriminately with all sgRNA.

To overcome the limitations of the fluorescently tagged Cas protein system, the fluorescent sgRNA system was recently developed [204]. Compared to the fluorescently tagged dCas13 system, the fluorescent sgRNA system labels the sgRNA with fluorescent dyes instead of fusing dCas13 with fluorescent protein (Figure 6d). In live cells, non-assembly, no-target, and off-target sgRNA is degraded rapidly. Conversely, on-target sgRNA is strongly protected within the Cas9:gRNA:DNA ternary complex from ribonuclease degradation [204,207]. Thus, the fluorescent sgRNA system provides low background noise from non-assembly, no-target, and off-target fluorescent sgRNA. In addition, this system is amenable to multiplexing since the fluorescence is labeled on the sgRNA. However, like the fluorescently tagged Cas protein system, there is still a significant amount of effort required to optimize the guide sequence of the sgRNA and the potential risk of disrupting other RNA binding proteins of target RNA. In conclusion, the distinct approaches of live-cell RNA single-molecule tagging each have their own advantages and limitations. Therefore, the utilization of multiple RNA tagging approaches would allow researchers to have fundamentally deeper and more comprehensive understanding of RNA export.

## Data Availability

Data supporting this review are published and available at the Proceedings of the National Academy of Sciences https://doi.org/10.1073/pnas.2015621118
